# Acknowledgement to Reviewers of *Metabolites* in 2019

**DOI:** 10.3390/metabo10010041

**Published:** 2020-01-17

**Authors:** Metabolites Editorial Office

**Affiliations:** MDPI, St. Alban-Anlage 66, 4052 Basel, Switzerland

The editorial team greatly appreciates the reviewers who have dedicated their considerable time and expertise to the journal’s rigorous editorial process over the past 12 months, regardless of whether the papers are finally published or not. In 2019, a total of 318 papers were published in the journal, with a median time to first decision of 18 days and a median time from submission to publication of 40 days. The editors would like to express their sincere gratitude to the following reviewers for their generous contribution in 2019:
Abu-Reidah, Ibrahim M.Lyakhova, Ekaterina G.Achyuthan, Komandoor E.M Kamal, Abu HenaAdam, TomášMa, XiaochaoAdamski, JerzyMaack, ChristophAeddula, Narothama ReddyMacKinnon, NeilAfford, SimonMAHAR, ROHITAhmed, IshfaqMahieu, Nathaniel G.Ahn, TaehoMahieu, Nathaniel G.Alexandre-Gouabau, Marie-cécile F.Maioli, MargheritaAlexeyev, MikhailMaitre, LeaAlgar, ElizabethMalaguarnera, GiuliaAlizadeh, HosseinMallela, Shamroop KumarAllen, DougMancinelli, RominaAltraja, AlanMandard, StéphaneAmato, JussaraManfredi, Kirk P.Ana, Fernández-OcañaManfredini, StefanoAndersen, Maria KarolineMangalagiu, IonelAndreescu, NicoletaMannella, ValeriaAnedda, RobertoMarcel R., De ZoeteAnna, Stanislawska-SachadynMarco, FazzariAon, Miguel A.Mardinoglu, AdilAquilano, KatiaMarhuenda-Egea, Frutos CarlosAraujo, PedroMaria Irene, BelliniArgmann, Carmen A.Marin, LuminitaArita, MasanoriMarina, EvichArita, MasanoriMarkuszewski, MichałArtegoitia, Virginia M.Markuszewski, MichelBabickova, JankaMartano, GiuseppeBąchor, RemigiuszMartin, KrssakBackman, TylerMartínez, José LuisBahrim, GabrielaMartini, Sharyl R.Bain, JamesMartins, IsabelBairaktari, EleniMartín-Sõmer, AnaBais, PreetiMashima, RyuichiBakillah, AhmedMasui, KentaBalunas, Marcy J.Mathe, EwyBanci, LuciaMatsuda, FumioBanerjee, SudipMattila, IsmoBani, PaoloMayengbam, ShyamchandBankova, VassyaMayeuf-Louchart, AliciaBarberini, LuigiMayo-Prieto, SaraBarlow, ChristopherMc Auley, Mark T.Barreca, DavideMcCall, Jennifer R.Barros, AntonioMcConville, MalcolmBasit, AbdulMcGeachie, MichaelBeale, DavidMcgregor, NeilBeckett, EmmaMei, YangBeger, RichardMeleady, PaulaBehrends, VolkerMerchant, AndrewBelbahri, LassaadMermier, Christine MBeltowski, JerzyMerritt, MatthewBeniddir, MehdiMesaros, Clementina A.Benina, MariaMesnard, FrançoisBernhoft, AkselMeyer, JesseBernillon, StéphaneMézes, MiklósBertram, HanneMiller, MarcusBian, ZhaoxiangMillet, ÓscarBišová, KateřinaMilutinović, Bojana S.Blackburn, GavinMinocha, Subhash C.Board, MaryMiro, JordiBobe, GerdMishur, Robert J.Boccard, JulienMisra, Biswapriya BiswavasBock, ChristianMitchell A, SullivanBöcker, SebastianMittapelly, PriyankaBorghi, ElisaMiura, ShinjiBoros, LaszloMoestue, Siver A.Bos, Lieuwe D.Mohajeri, M. HasanBose, Jeffrey L.Moholdt, TrineBoselli, EmanueleMoing, AnnickBowden, John AlfredMok, Daniel Kam-WahBowen, BenjaminMolinie, RolandBoyles, RichardMolins, Claudia R.Bozonet, StephanieMöller, GabrieleBrasili, ElisaMolognoni, LucianoBrebu, MihaiMonica, SandenBreves, GerhardMonk, JenniferBritz-McKibbin, PhilipMonsalve, MariaBroeckling, CoreyMontero, OlimpioBrown, Robert J.Moore, SteveBruce, ClintonMorales, Jose ManuelBrunetti, CeciliaMori, MatteoBrunius, CarlMorini, LucaBub, AchimMoseley, HunterBustamante, ClaudiaMoser, Ann B.Caboni, PierluigiMucci, AdeleCacciola, FrancescoMudura, ElenaCalado, Cecília R.C.Muhle-Goll, ClaudiaCaligiuri, IsabellaMukherjee, RajibCamañes, GemmaMulas, MaurizioCameron, JeffreyMuleo, RosarioCampana, OliviaMüller, ChristophCampanella, AlessandroMurata, JunCampbell, JaredMurphy, RachelCanela, NúriaMusco, NadiaCaney, SarahMusso, OrlandoCappelletti, GraziellaMuszyński, SiemowitCappello, TizianaMyridakis, AntonisCaraballo, MauricioNácher-Mestre, JaimeCarlson, RossNaghavi, MortezaCarmeli, ShmuelNair, SreenathCarnevale Neto, FaustoNajda, AgnieszkaCarrera, MónicaNajdekr, LukášCarriker, ColinNakagawa, HayatoCasado, NataliaNakagawa, TakashiCasey, JosephNambara, EijiCassol, EdanaNaponelli, ValeriaCavill, RachelNatsumeda, ManabuCespedes Feliciano, Elizabeth M.Naviaux, Robert K.Chacko, BaluNawade, BhagwatChadwick, Amy E.Nduhirabandi, FredericChagraoui, AbdeslamNeogi, UjjwalChaleckis, RomanasNeumann, SteffenChami, BelalNguyen, Canh HaoChandler, JoshuaNguyen Phuoc, LongChauchat, LucNicaud, Jean-MarcCheca, AntonioNikel, Pablo IvanChekmeneva, ElenaNishiyama, SoichiroChen, ChaoNiyazov, Dmitriy M.Chen, Jen-TsungNothias, Louis-FélixChen, Jing-HsienNowak, WieslawChen, ChiNowicki, MichałCheng, Leo L.Obanda, DianaChetwynd, AndrewOkumura, TadayoshiChitarrini, GiuliaOkunade, Adewole L.Chiu, Sheng-YiOlesti, EulaliaCho, Joo-YounOrenes Piñero, EstebanChoi, Young MinOresnik, Ivan J.Chorell, ElinOrso, EvelynChrist, BastienOse, JenniferChu, Su HOvermyer, KatherineChung-Davidson, Yu-WenOves-Costales, DanielCiaramelli, CarlottaPace, FabioCiborowski, MichalPacia, Marta Z.Cicero, NicolaPaglia, GiuseppeCiereszko, IwonaPalermo, AmeliaCimmino, GiovanniPalmer, Lachlan J.Coldea, TeodoraPanderi, IreneCollier, JasonPandey, Amit VCologna, StephaniePang, BoConsonni, RobertoPannkuk, EvanCooperstone, JessicaPapazian, StefanoCopié, ValerieParis, DeboraCordero, Chiara EmiliaPark, Sang WookCorl, Benjamin A.Park, SangkyuCornelison, Christopher T.Parkkila, SeppoCorsaro, CarmeloParry, Traci L.Cotrozzi, LorenzoPasqua, GabriellaCox, JamesPastor, VictoriaCroce, Anna CletaPatarrão, RitaCruickshank-Quinn, CharmionPathmasiri, WimalCuperlovic-Culf, MiroslavaPaton, ChadCuykx, MatthiasPatrice, ThierryCzamara, KrzysztofPauling, JoschDai, ZiweiPavagadhi, ShrutiDal Piaz, FabrizioPavlova, NatalyaDall’Acqua, StefanoPazos, FlorencioDanielak, DorotaPechlivanis, AlexandrosDar, Wasim A.Peirang, CaoDasenaki, MarilenaPeiretti, Pier GiorgioDash, RanjeetPembroke, J.TonyDaubon, ThomasPeng, Robert Y.David, LuminitaPereira, LeonelDe B. Harrington, PeterPereira, Ana LuisaDe Falco, BrunaPerestrelo, Rosa Maria De SáDe Jesus, Victor R.Pérez Trujillo, MíriamDe Santo, CarmelaPerła-Kaján, JoannaDe Seymour, JamiePerret, AlainDe Souza, David P.Pesek, JosephDeda, OlgaPessi, GabriellaDeigner, Hans-PeterPeter, StougaardDeja, StanislawPeters, KristianDel Coco, LauraPetkova, NadezhdaDerkach, AndriyPichler, IreneDettmer-Wilde, KatjaPinske, ConstanzeDi Filippo, MarziaPinu, Farhana R.Ding, Xiao-QiPiotto, M. MartialDing, LingPlavec, JanezDinis-Oliveira, Ricardo JorgePlaydon, MaryDionisio, GiuseppePlenis, AlinaDo, Duy NgocPluskal, TomášDolinsky, Vernon W.Postle, AnthonyDonaldson, GregoryPowers, RobertDonarski, JamesPozo, ÓscarDongiovanni, PaolaPrathipati, Pavan KumarDoria, EnricoPrieto Vidal, NataliaDunn, WarwickPromislow, DanielDuryee, Michael J.Pruvost, AlainEbbels, TimothyPuchalska, PatrycjaEdeas, MarvinPulavarti, Surya VSRKEghbalnia, Hamid RPuniya, Bhanwar LalEisenreich, WolfgangQazi, SohailEliseev, RomanQiu, YunpingEmami, PayamQuek, Lake-EeEngel, KathrinRadwan, MohamedEscribese, María M.Raftery, DanielEstall, JenniferRagusa, AndreaEvans, CharlesRamachandiran, IyappanEverett, JeremyRampanelli, ElenaFabregat Safont, DavidRango, MarioFairclough, Stuart JReinke, StaceyFaitot, FrancoisReyes-Garcés, NathalyFanizzi, Francesco PaoloReynoso, ElenaFanos, VassillosRidder, LarsFaria De Oliveira, MichelliRios-Covian, DavidFathi Abdallah, MohamedRivas, Mariella O.Fattuoni, ClaudiaRoager, Henrik MunchFenaille, FrancoisRoberts, MatthewFernandes, JolynRocchetti, GabrieleFernández-Ochoa, ÁlvaroRocha, MiguelFerrigno, AndreaRochfort, SimoneFilipe, LiuRodrigues, MárcioFlores, CarlosRogers, SimonFranceschi, PietroRolando, RebolledoFrankowski, MarcinRolle-Kampczyk, UlrikeFruhmann, PhilippRoscini, LucaFujishima, ToshieRosebrock, Adam PFukuda, TomohikoRoss, Robert L.Fukushima, AtsushiRöst, HannesFukushima, ArataRotili, DanteFuruya, TetsuyaRubert, JosepGajdoš, PeterRupasinghe, ThusithaGalkin, Alexander S.Saigusa, DaisukeGamir, JordiSaito, KosukeGanesan, PalanivelSalehzadeh-Yazdi, AliGao, BeiSalim, VonnyGarcía Fernández, AntoniaSalim, VonnyGarcia Martin, HectorSamaja, MicheleGarcia-Gimenez, DoloresSamczuk, PaulinaGarikipati, DilipŠamec, DunjaGašić, Uroš M.Šamec, DunjaGasparri, RobertoSamy, RaviGastaldelli, AmaliaSánchez-López, ElenaGaul, DavidSandulescu, MadalinaGawlik, AnetaSandusky, Peter OlafGerin, DonatoSansone, AnnaGhesquiere, BartSantacruz Ortega, Hisila del CarmenGil, AnaSantos López, Jorge ArturoGiles, CoreySantulli, GaetanoGiskeødegård, Guro F.Sarma, SauravGlaab, EnricoScalabrin, ElisaGlobisch, DanielScalbert, AugustinGogiashvili, MikheilSchaffer, AriGomes, NelsonSchedel, MichaelaGomez, Andres M.Schiefecker, Alois JosefGómez Castellà, CristinaSchlosser, GittaGómez Ocampo, IvánSchmidt, MarkGomez Roldan, Maria VictoriaSchrader, AndreaGoncalves, KuisSchripsema, JanGoncalves, OlivierSchulz, MargotGonda, SandorSchymanski, EmmaGonzález Domínguez, RaúlSciubba, FabioGonzález-Domínguez, RaúlSekula, PeggyGorska-Ponikowska, MagdalenaSemenov, IuriiGorynski, KrzysztofSen, ParthoGowda, NaganaSengupta, ArjunGraham, StewartSengupta, ArjunGramsbergen, JanSergi, DomenicoGrant, DavidShachar-Hill, YairGrasso, GiuseppeShang, ZhuoGreen, BrianShankar, EswarGreenway, StevenSharkey, Thomas D.Grinde, Maria T.Sheflin, AmyGrössl, MichaelShih, Pei-an BettyGuan, Xue LiShipley, PaulGuo, XinShoaie, SaeedGupta, Smiti V.Siadat, MaryamHaapala, EeroSilkina, AllaHagège, AgnèsSilva, Maria HelenaHageman, JosSilva, CatarinaHagemann, MartinSimeonov, VasilHalldórsson, SkarphéðinnSimirgiotis, MarioHampel, DanielaSimò, CarolinaHampel, DanielaSimón-Manso, YamilHampl, VladimírSinkkonen, JariHano, TakeshiSiopi, AikaterinaHaro, DiegoSirobhushanam, SirishaHart, Sarah R.Skibiński, RobertHarvey, PatriciaSleno, LekhaHashimoto, MakotoSlupsky, CarolynHawlitschka, AlexanderSolovyev, NikolayHayes, LawrenceSommerer, NicolasHedemann, Mette SkouSoni, DheerajHeinzmann, Silke SophieSorribas, AlbertHellmuth, ChristianSoufan, OthmanHerbst-Kralovetz, MelissaSpakowicz, DanHermann, GerritSprenger, HeikeHerminghaus, AnnaSquillaro, TizianaHernández, ÁngelSrivastava, SaurabhHibino, HiroshiSrivastava, SarikaHildenbrand, Sibylle L.Stanstrup, JanHoene, MiriamStark, Ruth E.Hoffman, JessicaStefanaki, CharikleiaHoffmann, Klaus H.Stefanon, BrunoHolt, Scott M.Stocchero, MatteoHong, YanjunStoskopf, MichaelHornburg, DanielStringer, Kathleen A.Hosmann, ArthurStuper-Szablewska, KingaHossain, EkramSturtevant, DrewHouzé, PascalSubashchandrabose, SureshHrabal, RichardSubramanian, ChitraHsiao-Wei, LiaoSugimoto, MasahiroHsu, Cheng-ChihSundekilde, Ulrik K.Hsu, Walter HawSunny, Nishanth E.Hu, YaxiSytykiewicz, HubertHuan, TianxiouSzacon, ElzbietaHuerta, BelindaSzymanski, JedrzejHumer, ElkeTada, YuichiHuszcza, EwaTakahashi, MasakiHuynh, KevinTam, Vincent C.Hwu, Wuh-LiangTapio, IlmaHyötyläinen, TuuliaTapissier, NathalieHysi, Pirro G.Tappia, ParamjitIjiri, DaichiTaraboletti, AlexandraIonete, Roxana ElenaTarantino, GiovanniIshikawa, TakahiroTaylor, MatthewIshimoto, TakahiroTayyari, FaribaIslam, M. NurulTebani, AbdellahIuga, DinuTeerds, Katja J.Ivanišević, JulijanaTenori, LeonardoIvanov, AlexanderTeschke, RolfJallet, DenisThaler, RomanJamwal, RohitashTheodoridis, GeorgiosJang, CholsoonThil Smith, Adam AlexanderJans, Judith J.M.Tikunov, Y. M.Jarret, Robert L.Timoshenko, AlexanderJaumot, JoaquimTimperio, Anna MariaJeltsch, AlbertTiziani, StefanoJeong, Tae-YongTolstikov, VladimirJeong, Keun-YeongTomaz, Cândida TeixeiraJeromel, AnaTomonaga, ShozoJiménez-Alemán, GuillermoToya, YoshihiroJohannsen, MogensTrainor, PatrickJohnson, CarolineTrammell, SamJones, DeannaTrengove, RobertJørgensen, KirstenTrippier, Paul C.Jurkonienė, SigitaTroisi, JacopoKachroo, PriyadarshiniTroncoso, A.M.Kalogiannis, StavrosTroncoso, AdrianKamlage, BeateTruong, Vi KhanhKaneko, GenTsugawa, HiroshiKant, SashiTsunoda, MakotoKarlic, HeidrunTu, ZhidongKartashov, AlexanderTzen, Jason T.Karu, NaamaUhle, FlorianKattner, LarsUngerfeld, E.M.Kayser, KlausUngurianu, AncaKechris, Katerina J.Usai, MariannaKeisaku, SatoUwiera, RichardKelly, RachelValentini, MassimilianoKendall, AlexandraValenzuela, Juan LuisKersten, RolandValerie B., O’LearyKhachik, FredValletta, AlessioKhadka, Manojvan dam, NicoleKiebish, MichaelVan Meulebroek, LievenKieliszek, MarekVeeravalli, VijayabhaskarKilk, KalleVelena, AstridaKim, SeonghoVemula, HarikaKim, Young-MoVenkat R, PannalaKim, Geun-BaeVergara, FreddKimber-Trojnar, ŻanetaVerhoeven, AswinKind, TobiasVermathen, PeterKindt, AlidaViana, FlaviaKirwan, JenniferVidal, ArnauKobuchi, ShinjiVidican, RoxanaKocira, SławomirVilela, AliceKokotos, GeorgeVillaseñor, AlmaKorenblum, ElisaVirginio Agostoni, CarloKosinsky, Robyn LauraVirtanen, RistoKot, Anna MariaVo, GiauKoulman, AlbertVon Eckardstein, ArnoldKraus, DanielVoy, BrynnKremling, AndreasWahi, KanuKrug, SebastianWahli, WalterKruger, Nicholas J.Waldherr, SteffenKrumsiek, JanWaldmeier, FelixKrumsiek, JanWalker, HeatherKudłak, BłażejWalsh, AaronKues, Wilfried A.Wang, ZenengKulbacka, JulitaWang, San-YuanKuligowski, JuliaWang, PengchengKumar, PraveenWang, YifeiKurka, OndrejWang, GuanKuś, PiotrWang, KunKuzma, MarekWang, ZhenKwon, Sung WonWard, LeighLa Barbera, GiorgiaWase, NishikantLa Rosa, CarmeloWatanabe, MikiLaghi, LucaWatrous, JeramieLaiakis, EvageliaWatson, Aaron M.Lambert, JoergWatson, DavidLamichhanea, SantoshWei, WeiLanning, NathanWeinhold, AlexanderLau, Chung-Ho EsmondWeljie, AalimLauro, Maria RosariaWesterhuis, Johan A.Le Gall, GwenaelleWeston, Leslie A.Le Roy, CarolineWeston, PaulLee, Jin-YongWheelock, CraigLee, Do YupWhite, PhillipLee, Hye SukWienkoop, StefanieLei, ZhentianWietrzyk, JoannaLeimanis, MaraWilson, PhilippeLeon, CarlosWilson, ThomasLi, GuohuiWińska, KatarzynaLi, YanWiwie, ChristianLi, QianshengWojtyla, ŁukaszLi, DongyingWong, AlanLi, FengWood, Troy D.Liang, Jessie QiaoyiWöstemeyer, JohannesLiebeke, ManuelWu, Hung-MingLim, EdwinWu, DavidLin, Ying-ChiXia, JianguoLin, QishanXiao, ZhengtaoLionetto, Maria GiuliaYamada, SohsukeLisec, JanYamashita, AtsushiLiu, YongliangYang, Wei-hsiungLiu, ZhiqianYasuda, KazukiLiu, Jun-HongYeh, HeidiLocci, EmanuelaYehualaeshet, TeshomeLodge, John K.Zaini, PauloLoftfield, ErikkaZamyatnin, AndreyLogan-Sprenger, Heather M.Zeeshan, MuhammadLomonaco, TommasoZelezniak, AleksejLongo, ValentinaZhang, BoLongo, RoccoZhao, LiangLorkiewicz, Pawel K.Zhou, LeiLu, Chi-YuZhou, MiLucarelli, GiuseppeZieliński, JacekLuccini, LuigiZiemann, EwaLuchinat, ClaudioZienkiewicz, AgnieszkaLuke, MarneyZou, ChunbinLundeheim, NilsZuk, MagdalenaLuo, XianŽupanič, AnžeLusczek, Elizabeth R.Zuther, Ellen

